# Morphine versus Nalbuphine for Open Gynaecological Surgery: A Randomized Controlled Double Blinded Trial

**DOI:** 10.1155/2014/727952

**Published:** 2014-04-14

**Authors:** Shiv Akshat, Rashmi Ramachandran, Vimi Rewari, Chandralekha, Anjan Trikha, Renu Sinha

**Affiliations:** Department of Anaesthesiology, All India Institute of Medical Sciences, Ansari Nagar, New Delhi 110029, India

## Abstract

*Introduction.* Pain is the commonest morbidity after open surgical procedures. The most effective treatment of postoperative pain is opioid therapy. Morphine, the commonly used opioid, is associated with many side effects including respiratory depression, sedation, postoperative nausea vomiting, and pruritus. Nalbuphine, on the other hand, is known to cause less respiratory depression. Thus this study was undertaken to compare the intraoperative and postoperative analgesic efficacy and side effect profile of the two drugs. *Methodology.* 60 patients undergoing open gynaecological surgery were randomized to receive either morphine (Group M) or nalbuphine (Group N) in the intraoperative and postoperative period. Intraoperative analgesic efficacy (measured by need for rescue analgesics), postoperative pain by visual analogue scale, and side effects like postoperative nausea, vomiting, sedation, respiratory depression, and pruritus were compared in both groups. Intraoperative and postoperative heart rate and blood pressure were also compared between the groups. *Results.* Need for intraoperative analgesia was significantly more in Group N (*P* = 0.023). Postoperative VAS scores were significantly different between the groups at various time points; however, none of the patients required any rescue analgesia. The incidence of various side effects was not significantly different between the groups. The haemodynamic profile of patients was comparable between the groups in both intraoperative and postoperative period. *Conclusion.* Nalbuphine provides less effective intraoperative analgesia than morphine in patients undergoing open gynaecological surgery under general anaesthesia. Both drugs, however, provided similar postoperative analgesia and had similar haemodynamic and side effect profile.

## 1. Introduction

Gynaecological surgeries like total abdominal hysterectomy, vaginal hysterectomy, and ovarian tumour resection are common surgeries performed in adult female population throughout the world. Although there is an increasing trend towards laparoscopic surgeries, open procedures continue to remain common therapeutic modalities especially in the developing countries. Pain is reported more commonly in patients undergoing open procedures than laparoscopic procedures [1, 2].

Postoperative pain and tissue injury associated with surgery initiate a systemic stress response [3] which has neuroendocrine, immunological, and haematological responses.

Opioids are an important modality of postoperative pain management. They blunt the neuroendocrine stress response to pain [4]. Morphine is the most common opioid used for postoperative analgesia. However it is associated with several adverse effects like respiratory depression, nausea, vomiting, pruritus, constipation, urinary retention, bradycardia, and hypotension. In view of the side effects, various other agents like tramadol, buprenorphine, NSAIDS, and paracetamol have been used, but morphine continues to remain gold standard.

Nalbuphine, on the other hand, being mu antagonist and kappa agonist, has a ceiling effect in its respiratory depression [5, 6]; hence it is considered to be safer than morphine. Many studies have reported that incidence of adverse effects like pruritus and PONV is lower with nalbuphine in comparison with morphine [7–9].

The aim of this study is to compare the analgesic efficacy and side effect profile of morphine with nalbuphine, a mixed agonist antagonist in patients undergoing open gynaecological surgeries. The primary outcome measured was the postoperative scale (VAS) score in patients in the postoperative period. The secondary outcomes measured were the intraoperative rescue analgesic requirement and incidence of postoperative side effects.

## 2. Material and Methods

This study was conducted in the Department of Anaesthesiology of All India Institute of Medical Sciences, New Delhi, India, after approval from institute ethical subcommittee. Sample size was calculated after data was collected from 10 patients undergoing open gynaecological surgery with the anaesthetic technique mentioned above using morphine as the opioid analgesic. The mean VAS score at the time of shifting these patients to PACU was 3.5 with a standard deviation of 1.8. Keeping the power of the study as 80% and a 5% alpha error with a sample size ratio between two groups as 1, a total sample size of 52 was needed to identify a difference of 1 in the VAS score. Thus each group would require at least 26 patients. To allow for dropouts, we decided to include 30 patients in each group. Sixty ASA I and II patients, aged between 18 and 60 years, undergoing open gynaecological surgery were included in the study. The exclusion criteria were patient refusal, respiratory insufficiency, history of recent head injury and raised intracranial pressure, haemodynamic instability, abnormal thyroid function, administration of opioid in the last 4 hours, renal or hepatic insufficiency, history of any chronic pain, unstable personality and abuse liability, inability to comprehend PCA device, and history of chronic opioid use. This was a randomized double-blinded study. After routine preanaesthetic checkup, an informed written consent was taken from each patient. Before the surgery patients were shown a VAS of 0–10 for pain with 0 being no pain and 10 being worst pain ever felt. Patients were instructed upon the use of patient controlled analgesia (PCA) pump.

Patients were divided into two groups, that is, group M (*n* = 30), using morphine, and group N (*n* = 30), using nalbuphine, on the basis of drawing of an opaque sealed envelope. The anaesthesiologists managing the intraoperative and postoperative courses as well as patients were blinded to knowledge of the group to which they belonged. The drug solution to be used intraoperatively and postoperatively as PCA was prepared by an assistant by dissolving 20 mg of morphine or nalbuphine in 20 mL of normal saline to make 1 mg/mL concentration of opioid solution.

Patients were premedicated with 0.25 mg of oral alprazolam given on the night before and on the morning of surgery. Patients, after being shifted to the operation theatre, were subjected to monitoring of ECG, pulse oximetry, and noninvasive blood pressure.

Anaesthesia was induced with the unknown opioid in the dose of 0.1 mL/kg of the solution prepared, followed by a sleep dose of propofol, and muscle relaxation was achieved by vecuronium (0.1 mg/kg). Endotracheal intubation was done after 3 minutes of bag and mask ventilation. Continuous monitoring of end tidal carbon dioxide was done after intubation.

Anaesthesia was maintained with isoflurane in oxygen and nitrous oxide to a MAC of 1–1.2. Boluses of vecuronium were used as and when required. The noninvasive blood pressure (BP), heart rate (HR), and oxygen saturation of blood (SpO_2_) were recorded every five minutes for the first 15 minutes followed by every 15 minutes. Intraoperatively, if analgesia was required, indicated by increase in HR or BP more than 20% of baseline, 0.05 mL/kg of the study group opioid was given. Rescue analgesia was given intraoperatively as fentanyl 1 mcg/kg, if the unknown opioid was deemed insufficient. The number of boluses of fentanyl needed was recorded.

Isoflurane was discontinued at the end of surgery. Neuromuscular blockade was reversed with neostigmine (50 mcg/kg) and glycopyrrolate (10 mcg/kg). The endotracheal tube was removed when patient met the extubation criteria: The Ramsay sedation score and side effects like nausea, vomiting, pruritus and respiratory depression (respiratory rate less than 8/min) were noted. Patient was shifted to postanaesthesia care unit (PACU) and a PCA machine was attached and postoperative analgesia was started by giving 3 mL bolus of the unknown opioid intravenously at the first complaint of pain (VAS > 3) by the patient. Thereafter patients were allowed to use PCA themselves. The settings of the PCA were 1 mL bolus with a lockout time of 10 min. There was no background infusion. The VAS, sedation, heart rate, noninvasive blood pressure, and respiratory and oxygen saturation were assessed at the time of shifting patient to PACU, 1/2 hour, 1 hour, 2 hours, 6 hours, 12 hours, and 24 hours after shifting patient to PACU. Thereafter, patients were shifted back to the wards after routine instruction for postoperative analgesia.

### 2.1. Side Effects and Management

Patients were assessed for the following side effects.

*(1) Respiratory Depression*. It was defined as rate less than 8/min or SPO_2_ < 90%. Patient would have been assessed for supplemental oxygen therapy like CPAP, increasing inspired air oxygen concentration. PCA would have been stopped. Naloxone in incremental doses starting from 0.1 mg/kg to 0.5 mg/kg would have been given if respiratory depression did not respond to the above measures.

*(2) Pruritus*. Pruritus was defined by its presence or absence and was treated with reassurance. Diphenhydramine (25 mg) on demand was given, if reassurance was ineffective. If diphenhydramine failed to control pruritus, then infusion of naloxone at 1 mcg/kg/hr IV was used.

*(3) Nausea*. Nausea was noted by its presence; it was treated by ondansetron 4 mg IV. In case of persistence of symptoms 10 mg of intravenous metoclopramide was used.

*(4) Vomiting and Retching*. Vomiting was defined as forcible expulsion of gastric contents through oral route, while retching was the same as vomiting without actual expulsion of gastric content. The number of each episode was noted. The treatment was the same as that of nausea.

*(5) Insufficient Analgesia*. It was defined as VAS score ≥ 4 at rest during 24-hour period. Rescue analgesia in form of intravenous fentanyl 1 mcg/kg was given.

*(6) Hypotension*. It was defined as systolic BP less than 90 mm of Hg. Episodes of hypotension were treated with IV fluids and boluses of 3 mg ephedrine if needed.

## 3. Results

**Figure 1:**
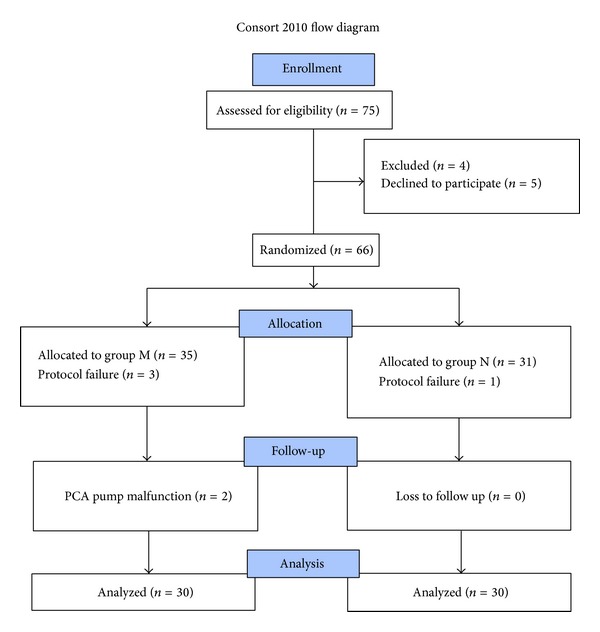
Flow of patients in the study.

The statistical analysis was done by using Stata 11.0 (College Station, TX, USA). The quantitative (nonparametric) data was analyzed by *t*-test (data being presented as mean ± standard deviation). The categorical (parametric) data was analyzed by chi-square test (data being presented as number (%)). Fisher's exact test was used wherever applicable. *P* value less than 0.05 was taken as significant. A total of 67 patients were screened for inclusion in the study. Flow of patients in the study is shown in Figure 1.

**Figure 2:**
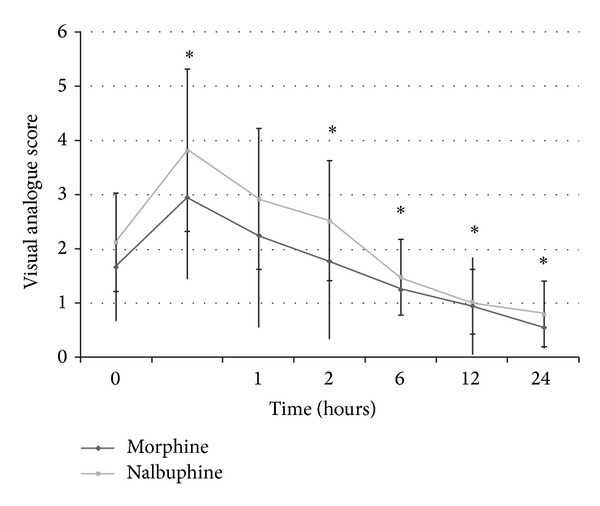
Visual analogue scale in the postoperative period in both groups. *Statistically significant difference between the groups. However, since the VAS was less than 4, patients did not require rescue analgesia.

The two groups were comparable with respect to age, weight, and ASA physical status (Table 1). The primary outcome, postoperative pain as measured by VAS, was significantly different between the two groups at various time points (Figure 2); however, none of the patients required any rescue analgesia in either of the groups in the postoperative period.

**Figure 3:**
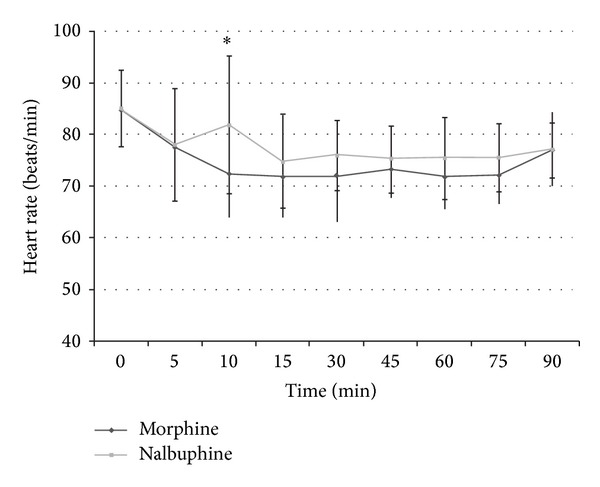
Heart rate in intraoperative period in both groups.

**Figure 4:**
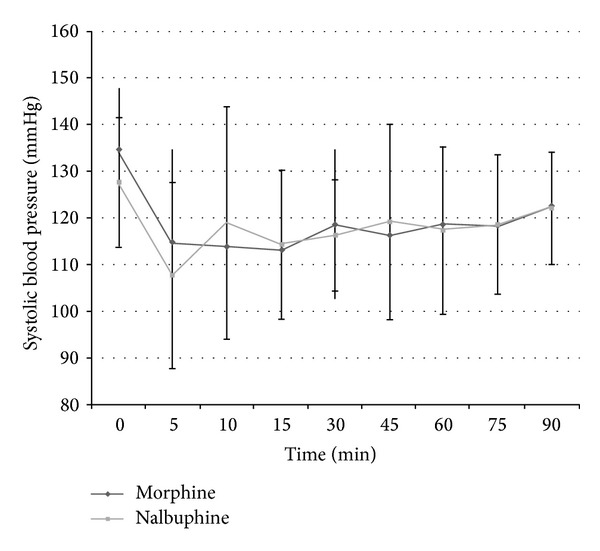
Systolic blood pressure in intraoperative period in both groups.

**Figure 5:**
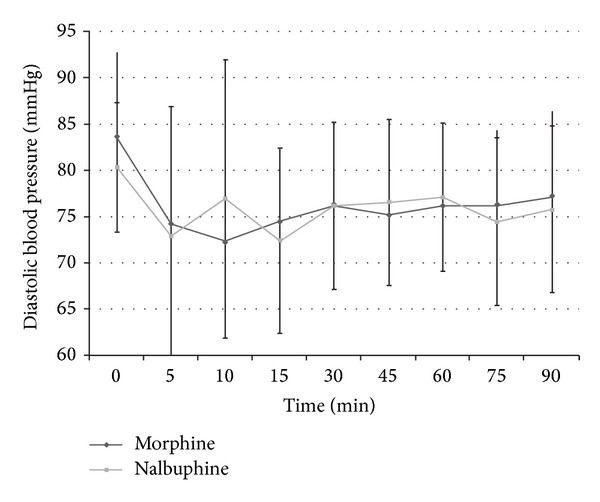
Diastolic blood pressure in intraoperative period in both groups.

**Figure 6:**
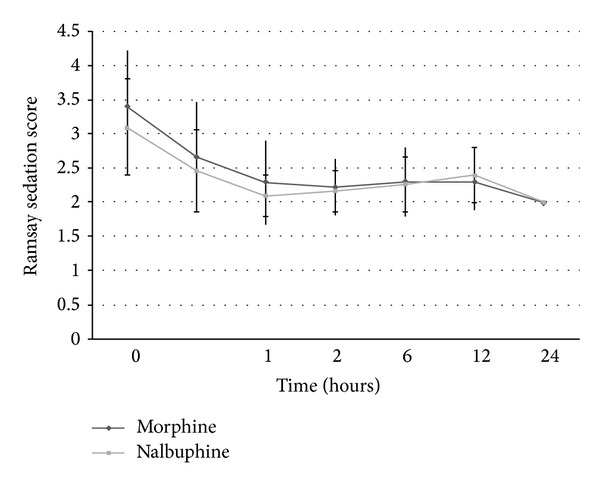
Ramsay sedation scale of patients in the postoperative period in both groups.

The haemodynamic parameters were monitored intraoperatively till the end of surgery. However statistical evaluation was done only for 90-minute duration intraoperatively as only 7 cases in both groups progressed beyond 90-minute duration and meaningful statistical comparison would not have been possible for duration beyond 90 minutes. The intraoperative heart rate and systolic and diastolic blood pressure trends in the two groups are shown in Figures 3, 4, and 5. Need for intraoperative rescue analgesia was significantly different between the two groups (Table 2). Postoperative sedation scored with Ramsay sedation score is shown in Figure 6. Postoperative opioid related side effects are shown in Table 2. Postoperative haemodynamic parameters were not different between the two groups.

No patient in either group had any episode of hypotension or respiratory depression in the postoperative period.

## 4. Discussion

This study was undertaken to compare the analgesic efficacy of nalbuphine (a kappa agonist) with morphine (predominantly a mu agonist) in female patients. Many previous studies have studied the possibility of opioids being more efficacious in females than in males. A previous study by Gear et al. in 1999 found this property of sexual dimorphism to be more prominent for kappa agonists, that is, nalbuphine and butorphanol [10]. Hence keeping this in mind the study was undertaken in female population only. The analgesic potency of nalbuphine is equivalent to that of morphine on a milligram basis. Thus equivalent doses of both drugs were chosen for both intraoperative (0.1 mg/kg) and postoperative analgesia (PCA settings of 1 mg bolus, lockout time of 10 minutes, without any background infusion). In our study we found that the number of patients requiring additional analgesia intraoperatively was significantly more in nalbuphine group (5 as compared to 0 in morphine group). This is in contrast to previous studies where analgesic efficacy has been seen to be equivalent. Minai and Khan [8] reported that the need for supplemental analgesia was lower with patients in nalbuphine group than with patients in morphine group. This can be explained on the basis of higher dose of nalbuphine used in their study (0.2 mg/kg as compared to 0.1 mg/kg of morphine). On the other hand, increasing the dose of nalbuphine above has been shown not to provide improved analgesic efficacy. In a volunteer based experimental study analgesic efficacy of morphine and nalbuphine was compared in 6 male patients subjected to pain and a ceiling effect in analgesic efficacy of nalbuphine in doses above 0.15 mg/kg was found [5]. There is a possibility that for surgical pain as in our study the clinical efficacy may be lower with nalbuphine due to ceiling effect seen in its analgesic efficacy. Inadequate surgical analgesia has been reported in previous 2 studies as well [11, 12].

Nalbuphine has complex pharmacodynamics with agonism at kappa receptors and antagonism at mu receptors. The analgesia via kappa receptors reaches a ceiling effect [5, 6] and thus provides unpredictable analgesia for surgical procedures. Thus the analgesic effect of nalbuphine is not pharmacokinetically predictable and rather depends on its complex pharmacodynamic profile. In the postoperative period although statistically significant difference in VAS scores was seen at various time points, treatment failure resulting in need for rescue analgesia was never seen. Thus it can be postulated that nalbuphine may be adequate for postoperative pain with these PCA settings. Yeh et al. [7] in a study using different combinations of morphine and nalbuphine found no difference in PCA requirements in postoperative period in patients undergoing open gynaecological surgeries. The PCA setting used in this study was similar as in our study with equivalent doses of morphine and nalbuphine. Cahalan et al. [13] have also described effective pain relief in posthysterectomy patients using the same settings, that is, 1 mg bolus with 10-minute lockout time using nalbuphine.

In general many other studies have also described adequate postoperative analgesia after varied surgical procedures in various population groups using nalbuphine either in intravenous or intramuscular regimes [9, 14–16].

The study also compared intraoperative heart rate and systolic and diastolic blood pressure in both groups. The statistically significant rise at 10 minutes in nalbuphine group can be explained by the inadequate analgesia obtained in 5 patients at the start of surgery which may have affected the overall mean haemodynamic values. Morphine is also known to cause bradycardia, probably by stimulation of vagal nuclei in medulla and direct depressant action on sinoatrial node, especially when coadministered with volatile anaesthetic agents [13]. Lake et al. have also reported less cardiac depression with nalbuphine in comparison to morphine, even when the former is used in high doses (3 mg/kg) in cardiac surgeries [17].

Our study also compared side effect profiles of both drugs. Morphine is a pure agonist whereas nalbuphine is a partial agonist. Morphine has an agonist action on all opioid receptors whereas nalbuphine is kappa agonist and weak mu antagonist. Hence, morphine has both spinal and supraspinal components in its analgesic effect whereas nalbuphine has predominantly spinal components. Respiratory depression caused by nalbuphine has a ceiling effect at higher doses. Morphine causes pruritus, whereas nalbuphine does not share this side effect. In fact nalbuphine may be used to treat morphine induced pruritus. Other side effects and pharmacodynamic profile are similar between these two drugs. In our study, sedation scores were comparable in both groups immediately after shifting to PACU. Sedation decreased with time in both groups and was statistically comparable in both groups throughout the PACU stay. Fragen and Caldwell who used either 0.1 mg/kg of intravenous morphine or nalbuphine as premedication, 11 to 14 minutes before induction of anaesthesia, also reported sedation to be comparable between both groups [9]. Ho et al. compared PCA nalbuphine or PCA morphine with a bolus dose of 1 mg and lockout time of 10 minutes, for postoperative analgesia after abdominal hysterectomy or myomectomy under spinal anaesthesia [14]. They also reported sedation to be statistically comparable between the two groups. Minai and Khan [8] reported that time interval between reversal of neuromuscular paralysis and patient's ability to tell name is greater with patients of nalbuphine group (16.2 min) than those of morphine group (11.2 min) in their study. However this again may be due to higher dose of nalbuphine used in this study.

The incidence of pruritus was more in morphine group (2 versus 0) although the difference did not reach statistical significance. Pruritis is a common side effect of use of opioids and occurs via* agonism* at mu receptors. Nalbuphine, on the other hand, is an* antagonist* at mu receptors and thus does not cause any pruritis. Absence of pruritus with nalbuphine has also been reported by other authors [7, 8]. Mechanism of PONV after use of opioids is not exactly known. Morphine is known to cause more PONV (48%) than nalbuphine (36%) [18]. In our study the difference was there (9 versus 6), but no statistical significance could be shown. We did not observe respiratory depression in any of the patients in postoperative period. Similar findings have been seen in various studies [7, 14–16, 19].

In summary nalbuphine was found to be less efficacious for intraoperative analgesia than morphine in equivalent doses in patients undergoing open gynaecological surgery. The postoperative analgesic efficacy of both drugs when used in PCA in equivalent doses of both drugs was similar. No patient in nalbuphine group had pruritus whereas two patients in morphine group had pruritus. The incidence of sedation and postoperative nausea and vomiting was similar in both groups.

## Figures and Tables

**Table 1:** Demographic data.

Parameters	Group M	Group N	*P* value
Age in years (mean ± SD)	40.20 ± 11.49	41.37 ± 12.35	0.706

ASA physical status (1/2)	10/30	7/30	0.399

Weight (Kg) (mean ± SD)	59.23 ± 10.55	54.37 ± 10.48	0.078

**Table 2:** Comparison between the two groups regarding the need for intraoperative analgesia and side effect profile.

Parameter	Group M	Group N	*P* value
Number of patients needing rescue analgesia intraoperatively	0 (0%)	5	0.023*

Number of patients having pruritus	2 (6.67%)	0 (0%)	0.492

Number of patients having PONV	9 (30%)	6 (20%)	0.476

*P value < 0.05 taken as significant.
